# Long-term performance evaluation of a novel 3.26 GBq ^68^Ge/^68^Ga generator

**DOI:** 10.1186/s41181-026-00429-z

**Published:** 2026-03-01

**Authors:** Xiaoyang Hu, Renxin Hu, Yao Yang, Yuping Li, Yan Zhao, Ning Liu, Qiang Ge, Shuang Zhang, Songdong Ding

**Affiliations:** 1 College of Chemistry, Sichuan University, Chengdu, 610064 China; 2 Chengdu New Radiomedicine Technology Co., Ltd., Chengdu, China; 3 Department of Nuclear Medicine, Affiliated Hospital of Southwest Medical University, Luzhou, China; 4 Institute of Nuclear Science and Technology, Sichuan University, Chengdu, China

**Keywords:** Radionuclide generator, Germanium-68, Gallium-68, ^68^Ge/^68^Ga generator, Performance evaluation

## Abstract

**Background:**

^68^Ga is a key radionuclide in PET due to its favorable decay properties, generator availability, and growing preclinical and clinical use of ^68^Ga-labeled radiopharmaceuticals. The performance of the ^68^Ge/^68^Ga generator is critical for efficient ^68^Ga utilization. Here, a novel modified TiO_2_-based ^68^Ge/^68^Ga generator (^68^Ge activity: 3.26 GBq) was evaluated for long-term performance, including ^68^Ga elution yield, ^68^Ge breakthrough, and metal impurities (Ti, Fe and Zn), and was verified in radiolabeling applications.

**Results:**

Over a two-year period, this generator maintained an average ^68^Ga elution yield of 79.8 ± 3.2% (*n* = 209, range: 70.8%–87.1%), an average ^68^Ge breakthrough as low as 0.00003% (*n* = 149, range: 0.0000035%–0.00031%), and Ti, Fe, and Zn concentrations well below the limit of 10 µg/GBq. The generator demonstrated compatibility with various precursors such as DOTA-TATE, Pentixafor and FAP-2286.

**Conclusion:**

The enhancement of the ^68^Ge(IV) loading capacity in the ^68^Ge/^68^Ga generator, along with improved ^68^Ga elution efficiency, facilitates the rapid and efficient production of ^68^Ga. This advancement contributes to the promotion and widespread adoption of ^68^Ga-based radiopharmaceuticals and benefits more patients.

**Graphical abstract:**

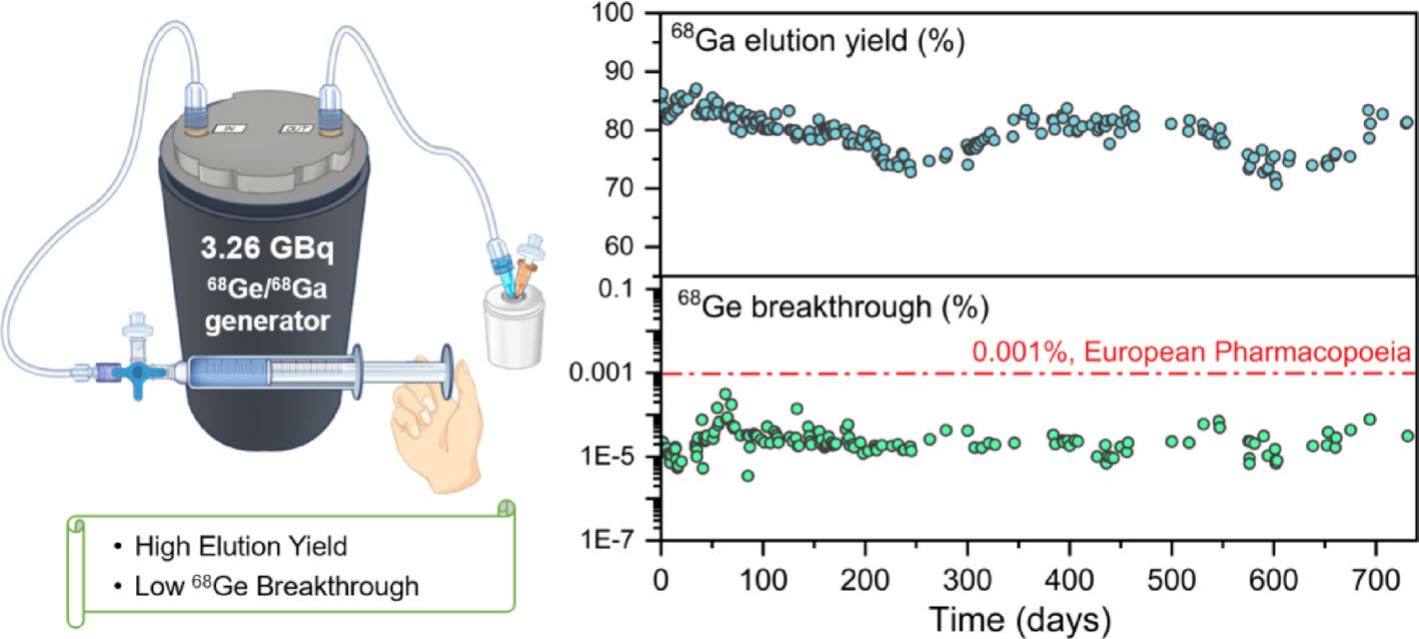

**Supplementary Information:**

The online version contains supplementary material available at 10.1186/s41181-026-00429-z.

## Background

The germanium-68(^68^Ge)/gallium-68(^68^Ga) generator has attracted increasing attention over the last couple of decades due to its important role in radiopharmaceuticals (Roesch 2012; Dash and Chakravarty 2019; Lepareur 2022; Mallapura and Eriksson 2025). ^68^Ga (half-life, 67.71 min) has become a routine clinical tool with well-established radiopharmaceuticals such as [^68^Ga]Ga-DOTATATE, [^68^Ga]Ga-DOTATOC and [^68^Ga]Ga-PSMA-11 (Dash and Chakravarty 2019; Lepareur 2022; Wang et al. 2024; Gavriilidis et al. 2025). The widely used ^68^Ge/^68^Ga generators are based on column matrices such as titanium dioxide (TiO_2_), stannic oxide (SnO_2_), pyrogallol-derivatized silicon dioxide (SiO_2_), and tantalum oxide (Ta_2_O_5_) (Velikyan 2015). Among them, the SnO_2_- and pyrogallol-derivatized SiO_2_-based generators have relatively high elution yields (> 80%) (Loc’h et al. 1980; Amor-Coarasa et al. 2016). However, the ^68^Ge breakthrough levels are relatively high (~ 0.001% for SnO_2_ and ~ 0.005% for pyrogallol-derivatized SiO_2_), which exceed the limit set by the European Pharmacopoeia (Ph. Eur. 11.0, 2464) of 0.001%, and the shelf lives of these generators usually do not exceed one year (Amor-Coarasa et al. 2016; Fialová et al. 2024). The TiO_2_-based generators, such as GalliaPharm^®^ (Eckert & Ziegler, Germany) and GalliAd^®^ (IRE Elit, Belgium), have very low ^68^Ge breakthrough (< 0.001%) and have been approved by the European Medicines Agency (EMA) and the American Food and Drug Administration (FDA). However, their initial elution yields are relatively lower than those of SnO_2_-based generators, generally about 70–75% (Roesch 2012; Dash and Chakravarty 2019; Sammartano et al. 2022). These above-mentioned generators more or less have incompatibilities in elution yield, ^68^Ge breakthrough, and shelf life. Furthermore, there are few studies on generators with ^68^Ge loading activities exceeding 1.85 GBq (50 mCi). In recent years, Isotopen Technologies Garching GmbH (ITG, Germany) has developed a 4.04 GBq (109.2 mCi) ^68^Ge/^68^Ga generator that maintains a very low ^68^Ge breakthrough. However, its elution yield is only approximately 60%, with a maximum of 65.2% (Waterhouse et al. 2020). This limits the effective utilization of the large quantity of loaded ^68^Ge, which is itself a costly radionuclide. Therefore, the development of novel ^68^Ge/^68^Ga generators with high ^68^Ge loading capacity and high ^68^Ga elution yield holds considerable significance for both research and practical applications.

Our group has developed a novel modified TiO_2_-based ^68^Ge/^68^Ga generator, with a ^68^Ge loading capacity of up to 3.26 GBq (88 mCi), herein referred to as the NRT ^68^Ge/^68^Ga generator (Chengdu New Radiomedicine Technology Co., Ltd., China). Through a long-term performance evaluation over two years, we systematically investigated the key performance parameters, including ^68^Ga elution yield, ^68^Ge breakthrough, and metal impurity levels (Ti, Fe and Zn). In addition, the applicability of the generator was confirmed through radiolabeling experiments conducted with several common precursors.

## Methods

### The ^68^Ge/^68^Ga generator

Hydrochloric acid (HCl, 30% Suprapur^®^, Merck KGaA, Germany) was diluted with 18.2 MΩ·cm water to prepare a 0.1 mol/L solution for generator preparation and elution. The modified TiO_2_ sorbent and NRT ^68^Ge/^68^Ga generator were produced by Chengdu New Radiomedicine Technology Co., Ltd. (China). The modified TiO_2_ sorbent was prepared by the sol-gel method (Macwan et al. 2011; Catauro et al. 2018), involving crystallization, heat treatment, grinding, sieving and washing. ^68^Ge stock solution was purchased from BWXT Medical Ltd. (Canada) and is present in dilute hydrochloric acid as germanium(IV) chloride. The radionuclidic purity of ^68^Ge is > 99.9%, and the radiochemical purity is > 99%. The content of each individual metal ion (Co, Cu, Fe, Nb, Ni, Pb and Zn) is < 1 µg/mCi, and that of Ga is < 2 µg/mCi. The 3.33 GBq (90 mCi) stock solution of ^68^Ge in a V-vial was diluted and loaded into the cold generator. Subsequently, the generator was washed with 0.1 M HCl. The activity of ^68^Ge loading in the generator is calculated by balancing the initial activity of the ^68^Ge stock solution and the residual activity of ^68^Ge found in the V-vial and single-use cassette. Finally, the generator was ready to elute one day after the ^68^Ge was loaded.

## Performance evaluation

The NRT ^68^Ge/^68^Ga generator was eluted with 5 mL of 0.1 M HCl. After each elution, the remaining HCl in the column was removed by passing air, known as the “dry” mode of operation (Romero et al. 2020). In addition, it can also be eluted using the “wet” mode of operation, in which the column remains filled with HCl until the next elution. After elution, the activity of ^68^Ga was measured immediately using a dose calibrator (VIK-202, Comecer SpA, Italy) and decay-corrected to the time of ^68^Ga elution (Eq. 1). The activity of the first elution each day was used to calculate the elution yield, allowing sufficient time for the in-growth of ^68^Ga. The elution yield was determined by dividing the decay-corrected measured eluted ^68^Ga activity by the ^68^Ga activity present on the column at the date and time of elution, as shown in Eq. 2:

1 $$ A_{{Ga}} = A_{0} \times k \times e^{{\lambda _{{Ga}} \times t_{1} }} $$

2 $$ ^{{{\mathrm{68}}}} {\mathrm{Ga}}\;{\mathrm{elution}}\:{\mathrm{yield}}(\% ){\text{ = }}\frac{{{\mathrm{A}}_{{{\mathrm{Ga}}}} }}{{{\mathrm{A}}_{{{\mathrm{Ge}}}} \times {\mathrm{e}}^{{ - \lambda _{{{\mathrm{Ge}}}} \times {\mathrm{t}}_{{\mathrm{2}}} }} }} \times {\mathrm{100}}\% $$

where *A*_Ga_ is the activity of ^68^Ga at the time of elution, *A*_0_ is the activity of ^68^Ga at the time of measurement. *k* is the calibration factor of the dose calibrator for ^68^Ga. *λ*_Ga_ represents the decay constant (*λ* = ln2/*T*_1/2_, *T*_1/2_ = 67.71 min) of ^68^Ga, and *t*_1_ is the time interval between the time of elution and the time of measurement. *A*_Ge_ is the activity of the ^68^Ge/^68^Ga generator at the calibration date. *λ*_Ge_ represents the decay constant (*λ* = ln2/*T*_1/2_, *T*_1/2_ = 270.95 d) of ^68^Ge, and *t*_2_ is the time interval between the time of elution and the calibration date of the ^68^Ge/^68^Ga generator.

The γ-ray spectrometry tests included the identification of principal γ-photons using a GR4020 high purity germanium γ-spectrometer (Canberra, USA). The ^68^Ge breakthrough in the eluate was assessed on a weekly basis following complete decay of ^68^Ga (> 48 h) and could be quantified by calculating the ratio of ^68^Ge activity to ^68^Ga activity in the eluate at the time of elution, as shown in Eq. 3:

3 $$ ^{{{\mathrm{68}}}} {\mathrm{Ge}}\:{\mathrm{breakthrough}}(\% ){\text{ = }}\frac{{{\mathrm{A}}_{{\mathrm{1}}} }}{{{\mathrm{A}}_{{{\mathrm{Ga}}}} }} \times {\mathrm{100}}\% $$

where *A*_Ga_ is the activity of ^68^Ga at the time of elution, and *A*_1_ is the activity of ^68^Ge in the eluate.

The metallic content in the eluates was determined using the inductively coupled plasma optical emission spectrometry (ICP-OES, iCAP PRO X, Thermo Scientific, USA). Other quality controls of the [^68^Ga]GaCl_3_ eluate were performed in accordance with the European Pharmacopoeia monograph (Ph. Eur. 11.0, 2464) (Nelson et al. 2022). Radiochemical identity and radiochemical purity (see Supplementary Information and Fig. S1) were determined using thin-layer chromatography (TLC) with a radio-TLC scanner (Mini-scan, Eckert & Ziegler, Germany). For visible particles, visual inspection was performed through a lead glass shield by using tongs to hold the sample vial against a light source, followed by gentle shaking to check for the presence of particulate matters. The pH values were measured using pH strips (pH range 0–6.0, MQuant^®^, Merck KGaA, Germany). The test for bacterial endotoxins was performed using the Limulus amebocyte lysate method (Gel-clot method: limit test. A limit test was adopted for direct comparison with parallel dilutions of a reference endotoxin by manual operation). Sterility was evaluated using the direct inoculation method (IAEA 2018). Inoculate a sample volume of the product directly into suitable culture media preferably fluid thioglycollate medium (Beijing Sanyao Technology Co., Ltd., China) and soybean-casein digest medium (Beijing Sanyao Technology Co., Ltd., China), followed by incubation at 30–35 °C and 20–25 °C respectively, for not less than 14 days. At intervals during the incubation period and at its conclusion, examine the media for macroscopic evidence of microbial growth.

## Radiolabeling

DOTA-TATE, Pentixafor and FAP-2286 were purchased from Nanchang Tanzhen Biological Technology Co., Ltd. (China). The ^68^Ga radiolabeling of DOTA-precursors was described previously (Nelson et al. 2022; Jia et al. 2025). First, prepare the reaction mixture by adding ^68^Ga eluate to a solution containing a suitable buffer (0.25 M sodium acetate solution), precursor, and any necessary additives. Next, incubate the mixture at 95 ˚C for 5–10 min to achieve ^68^Ga chelation. Then, purify the mixture using a Sep-Pak C18 light cartridge (Waters, USA), during which the ^68^Ga-labeled precursor is retained on the column while free ^68^Ga, impurities, and buffer are removed. Finally, elute the product by sequentially passing 1 mL of 50% ethanol solution and 4 mL of 0.9% NaCl solution through the column, and collect the eluate through a 0.22 μm sterile filter (ethanol max. 10% (*V*/*V*)). The quality controls of final product were performed using general methods (IAEA 2018; Nelson et al. 2022). Radiochemical purity was determined by analytical radio-HPLC (see Supplementary Information, Fig. S4, Fig. S5 and Fig. S6) and was quantified by integrating the area of the main peak corresponding to the ^68^Ga-labeled product, as well as the areas of the peaks corresponding to free ^68^Ga and all other undefined peaks with lower retention times. Radionuclidic purity and visible particles were evaluated using the same method as that used for the [^68^Ga]GaCl_3_ eluate.

## Results

**Fig. 1:**
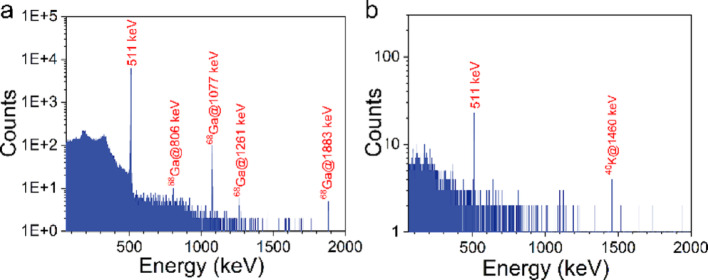
Gamma spectrum of [^68^Ga]GaCl_3_ eluate (a) and ^68^Ge in the eluate (b)

The NRT ^68^Ge/^68^Ga generator is based on a novel modified TiO_2_-based column matrix and was successfully loaded with 3.26 GBq (88 mCi) of ^68^Ge at the calibration date (May 16, 2023). The generator was eluted with 5 mL of 0.1 M HCl to prepare a [^68^Ga]GaCl_3_ solution with activity of 2.78 GBq (75.2 mCi), and the initial elution yield was 85.7%. The gamma spectrum of [^68^Ga]GaCl_3_ eluate is shown in Fig. 1(a). A series of peaks were observed, and the peak at 511 keV corresponds to the characteristic γ-ray emission from positron annihilation radiation. Peaks at 806 keV, 1077 keV, 1261 keV, and 1883 keV are all characteristic γ-rays of ^68^Ga. No significant peaks attributable to other radionuclidic impurities were detected. The radionuclidic purity of ^68^Ga, determined by peak area integration, was 100%. After 48 h and complete decay of ^68^Ga, the gamma spectrum of ^68^Ge in the eluate is shown in Fig. 1(b), it can be seen that the characteristic γ-rays of ^68^Ga were disappeared, and only the peak at 511 keV was obvious. In addition, the peak at 1480 keV corresponds to the characteristic γ-rays of ^40^K, a common peak in the natural background (Tomita et al. 2021). The activity of ⁶⁸Ge can be determined by measuring the 511 keV γ-ray peak corresponding to the decay of ^68^Ga in the obtained spectrum (Yüksel and Uğur 2022). The ^68^Ge breakthrough was calculated to be 0.000027%, based on the ratio of ^68^Ge activity to the ^68^Ga activity in the eluate at the time of elution (Eq. 3). Other quality controls of the [^68^Ga]GaCl_3_ eluate were listed in Table 1, the quality inspection results can comply with the Ph. Eur. acceptance criteria (2464) (Nelson et al. 2022).

**Table 1:** Acceptance criteria for the NRT ^68^Ge/^68^Ga generator

Test	Specification	Result
Appearance	Colorless, no particles	Colorless, no particles
Radionuclidic identity	62–74 min, 0.511 MeV and 1.077 MeV	Complies
Radiochemical identity	Rf value: 0-0.2	0.16
pH	0.5-2.0	1.0
Radionuclidic purity	≥ 99.9%	100.0%
Germanium-68	<0.001%	0.000027%
Radiochemical purity	≥ 95%	99.2%
Ti	≤ 10 µg/GBq	0.23 µg/GBq
Fe	≤ 10 µg/GBq	0.15 µg/GBq
Zn	≤ 10 µg/GBq	0.65 µg/GBq
Bacterial endotoxin	<35 EU/mL	<35 EU/mL
Sterility	Sterile	Sterile

**Fig. 2:**
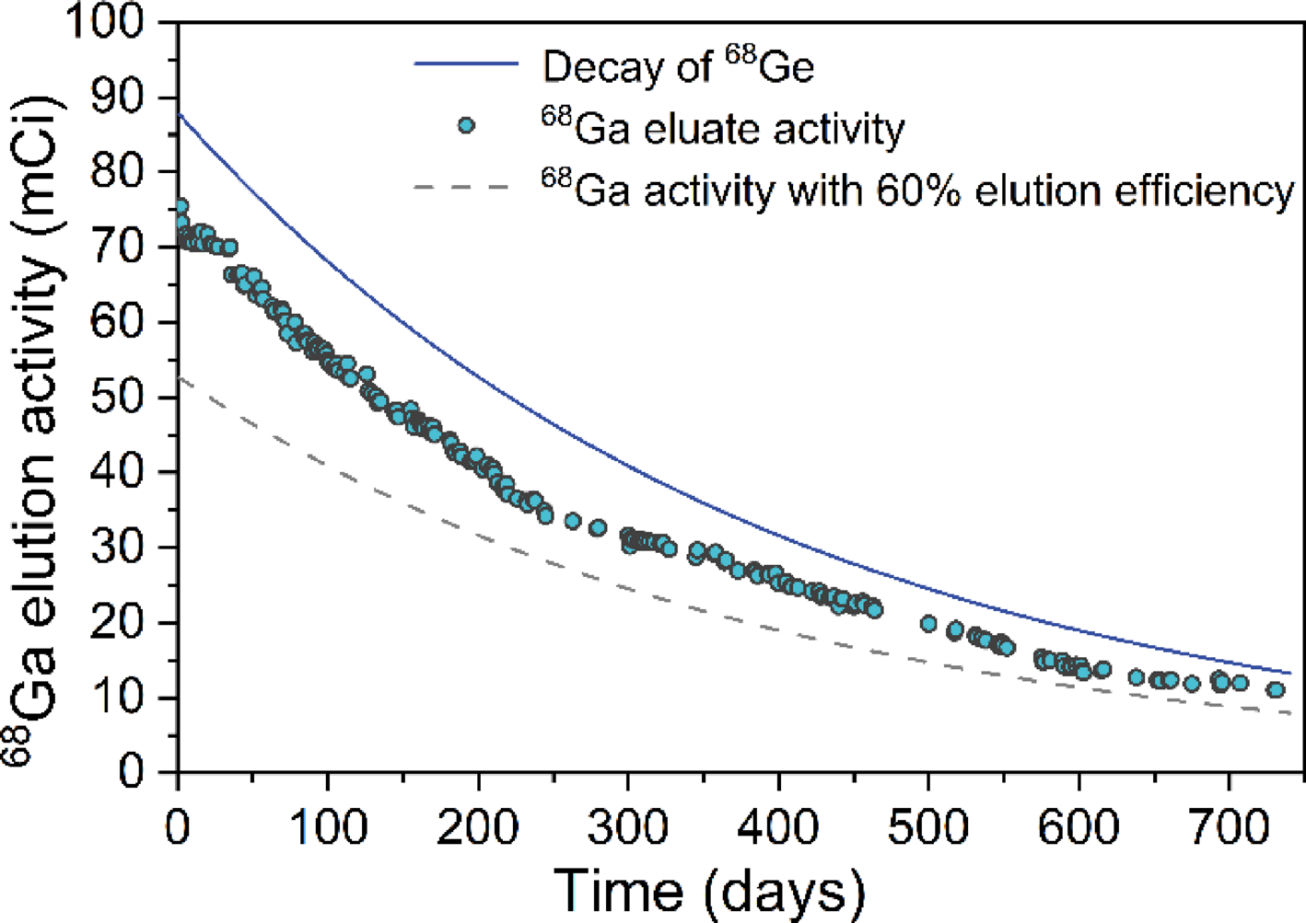
Long-term elution activity of the NRT ^68^Ge/^68^Ga generator

**Fig. 3:**
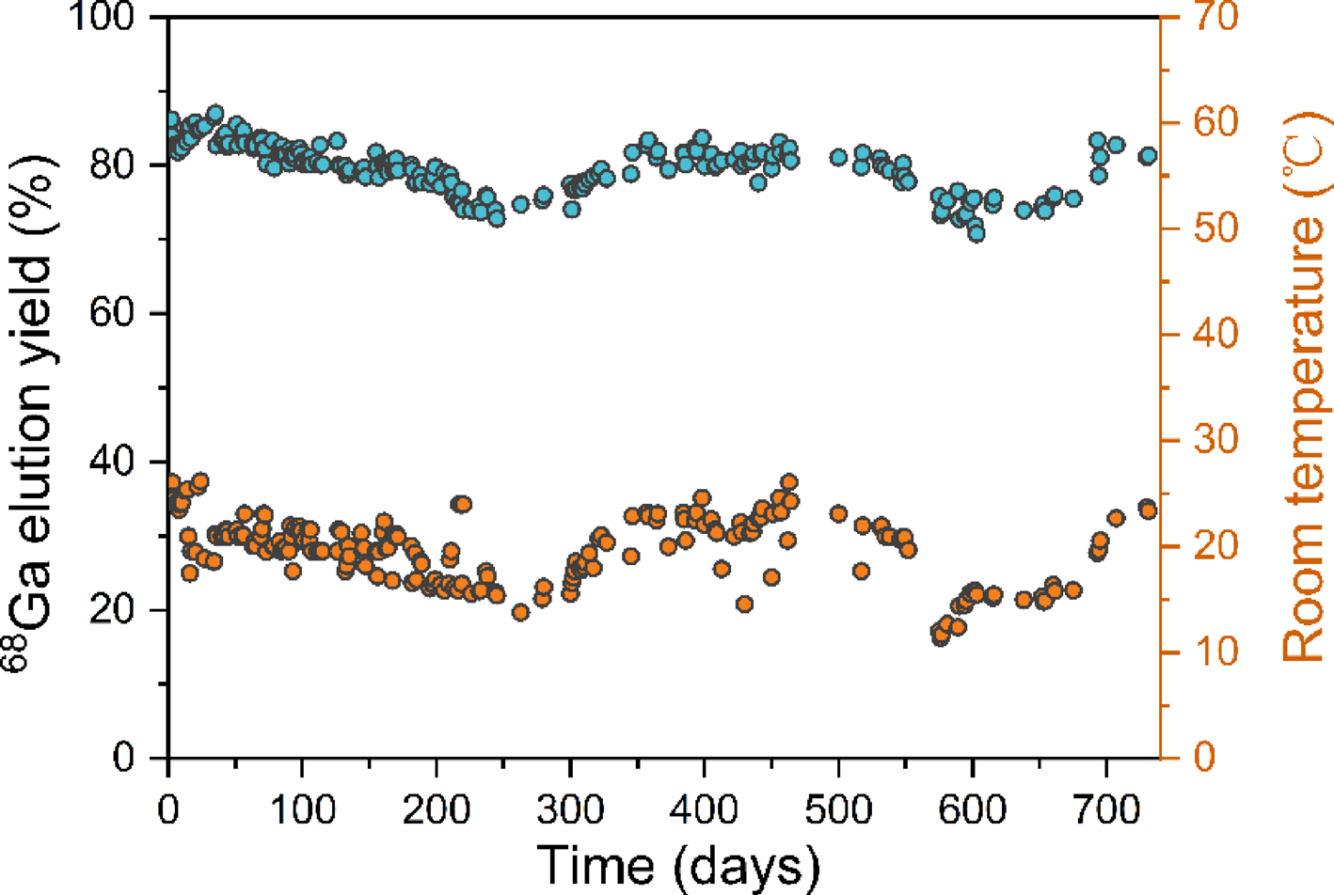
Long-term elution yield of the NRT ^68^Ge/^68^Ga generator at room temperature

The NRT ^68^Ge/^68^Ga generator was evaluated over an extended period of two years, totaling 232 elutions. Among them, 209 elutions represent the first elution of the day, and the rest represent the second or third elution of the day. Long-term elution activity of the NRT ^68^Ge/^68^Ga generator was shown in Fig. 2, and the blue line in the figure represents the decay curve of ^68^Ge over time. The initial activity of ^68^Ge was 3.26 GBq (88 mCi) at the calibration date (May 16, 2023), and the activity of ^68^Ge decreased to half after 271 days and to one quarter after 542 days. The grey dotted line indicates the theoretical ^68^Ga activity assuming a constant 60% elution yield for the ^68^Ge/^68^Ga generator, and thus decreases in parallel with the decay of ^68^Ge. Within the first 100 days after the generator was produced, the activity of ^68^Ga is high and closely follows the decay curve of ^68^Ge, indicating very high elution yield. As shown in Fig. 3, the ^68^Ga elution yield remained all above 80%. After 100 days, it gradually decreased. On the 245th day, it dropped to 72.8% at a room temperature of 15 °C. As the room temperature rose to 23 °C, the elution yield gradually recovered. On the 346th day, it returned to above 80%. Further observations indicated that seasonal temperature variations caused a recurrence of this trend. When room temperature fluctuated between 10 °C (11.4 °C, ^68^Ga elution yield: 73.3%) and 26 °C, the elution yield varied correspondingly. The average ^68^Ga elution yield was 79.8 ± 3.2% (*n* = 209) during the two-year evaluation, with a maximum of 87.1% (on the 35th day) and a minimum of 70.8% (on the 603rd day). The maximum elution interval was 35 days, during which no significant changes in elution yield were observed.

**Fig. 4:**
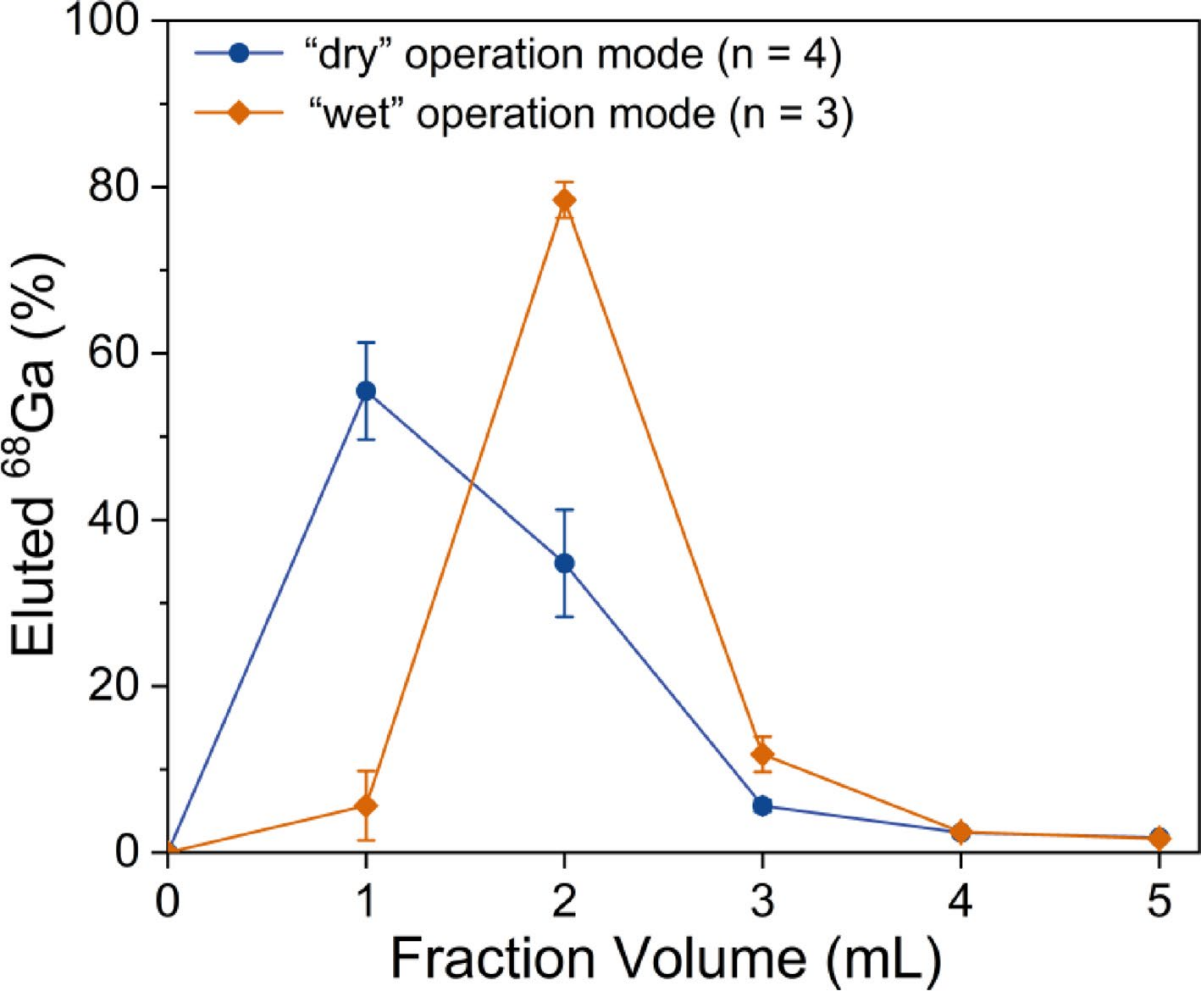
Elution profile study of the NRT ^68^Ge/^68^Ga generator using both the “dry” and “wet” operational modes

Figure 4 presents the elution profiles of ^68^Ga using both the “dry” and “wet” operational modes. Fractions of 1 mL were collected using 5 mL of 0.1 M HCl. In the “dry” mode, most of the ^68^Ga activity was eluted in fractions 1 and 2. The cumulative activity in the first 2 mL accounted for up to 90% of the total. In the “wet” mode, the majority of ^68^Ga was eluted in fractions 2 and 3. The first 1 mL can be discarded, and the following 2 mL, containing more than 90% of the total ^68^Ga activity, can be collected to obtain a higher activity concentration of ^68^Ga.

**Fig. 5:**
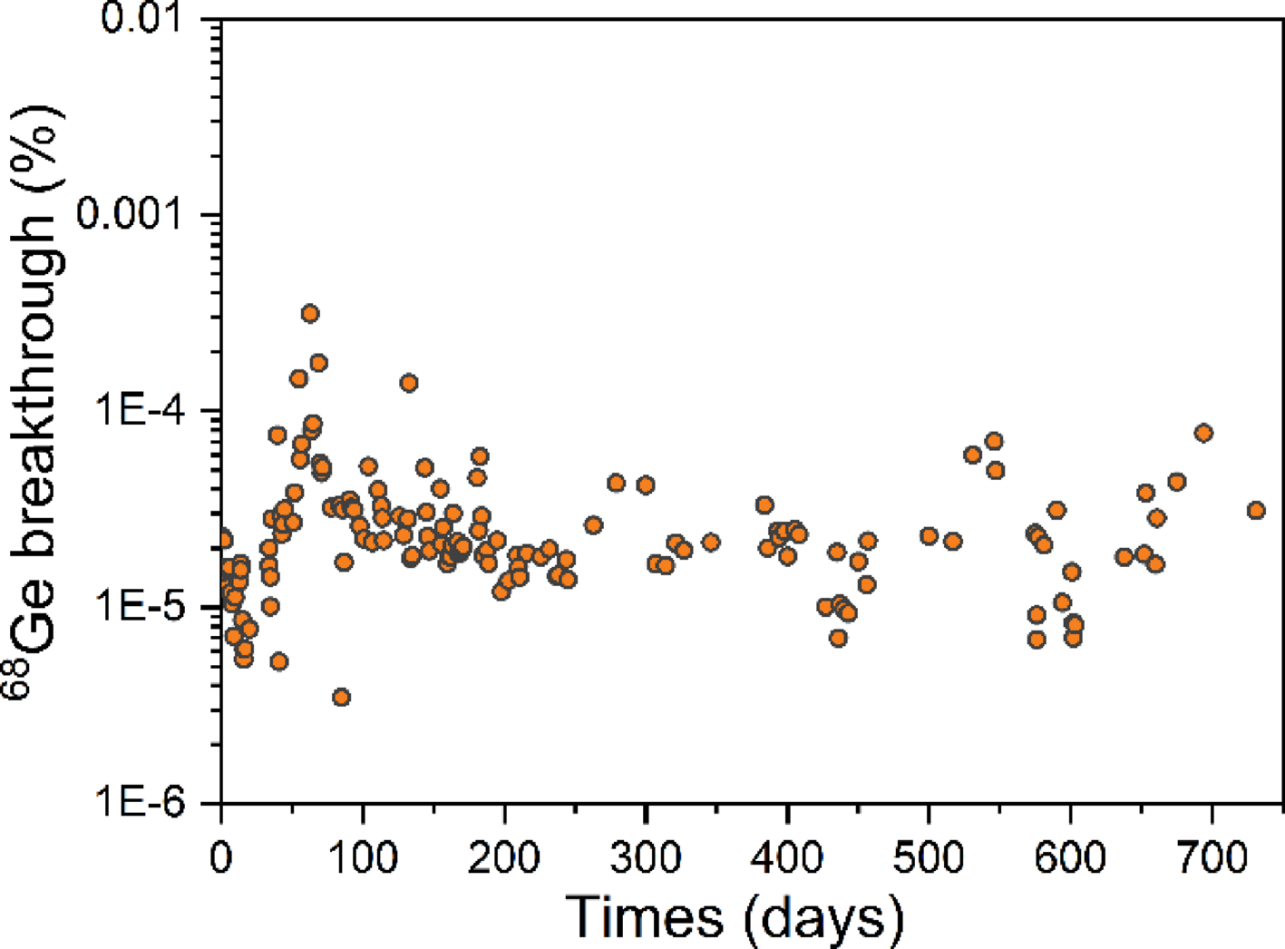
The long-term investigation of ^68^Ge breakthrough present in ^68^Ga eluates

**Fig. 6:**
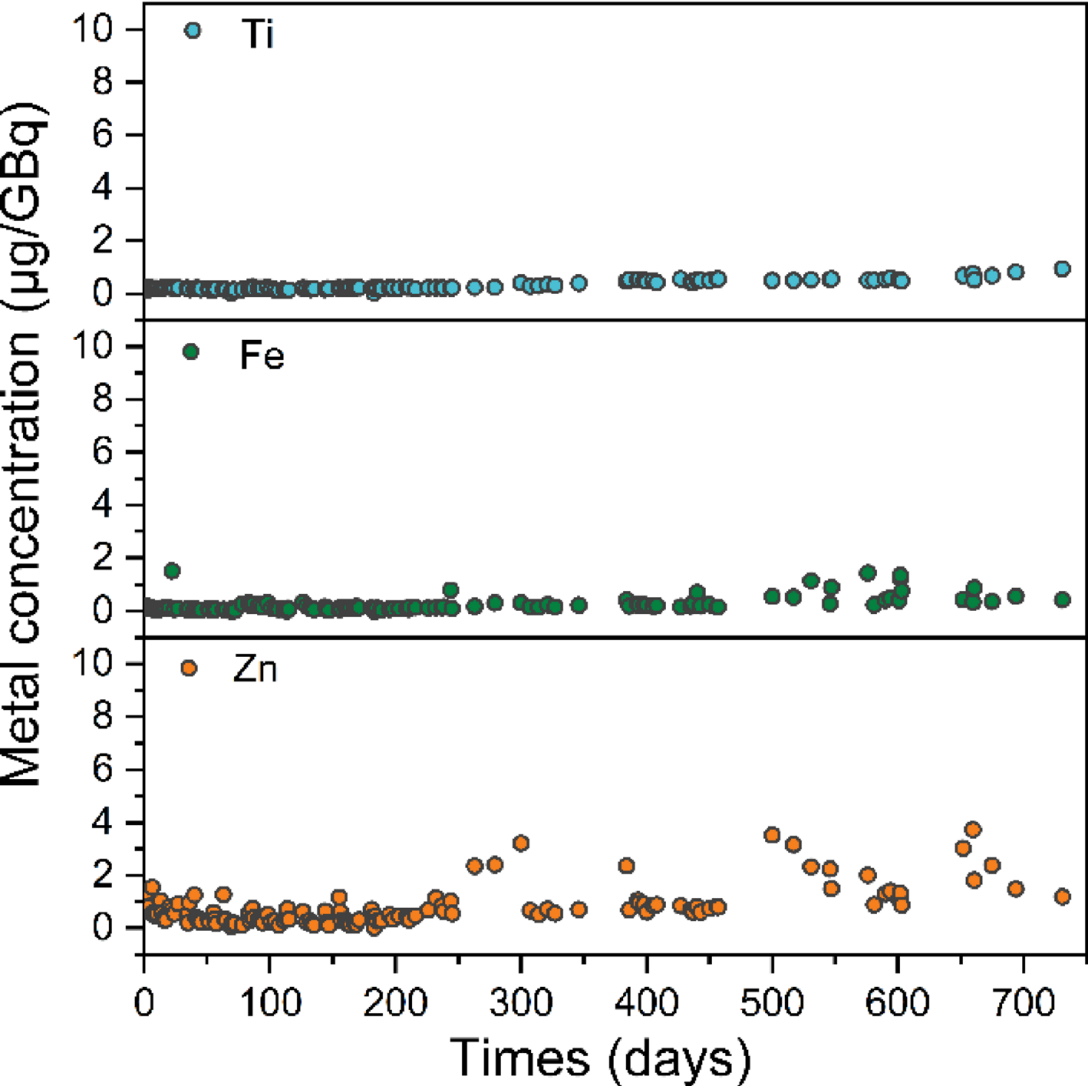
The long-term investigation of metal impurities (Ti, Fe and Zn) present in ^68^Ga eluates

The long-term investigation of the ^68^Ge breakthrough is presented in Fig. 5. Over the two-year evaluation period, the average ^68^Ge breakthrough was 0.000029 ± 0.000034% (*n* = 149), and the maximum value was 0.00031% (on the 63rd day), significantly below the 0.001% limit specified by the European Pharmacopoeia. Metal impurities have an important impact on ^68^Ga labeling. Therefore, a long-term analysis of various metal impurities (Ti, Fe, and Zn) in the eluate was conducted. As shown in Fig. 6, the concentrations of Fe and Ti remained very low. On the 731st day, the maximum Ti concentration reached 0.94 µg/GBq, demonstrating that the modified TiO_2_ material remained highly stable over a two-year period with negligible Ti release.

Zn is a specific metal impurity, as it is the decay product of ^68^Ga and can accumulate all the time. During the two-year evaluation, Zn levels ranged from 0 to 3.8 µg/GBq, rising to about 3 µg/GBq after 500 days. This increase may result from Zn accumulation in the column and a declining ^68^Ge activity (906 MBq at 500 days), possibly exacerbated by aging rubber components that release Zn. Despite the rise in later stages, Zn levels remained significantly below the European Pharmacopoeia limit of 10 µg/GBq. Furthermore, potential optimizations of the column system, particularly regarding the selection and formulation of rubber components, could further minimize the incidental introduction of Zn.

Radiolabeling of ^68^Ga with DOTA-TATE, Pentixafor and FAP-2286 was performed manually using a similar operating procedure, and the results were summarized in Table 2. It can be seen that the NRT generator is compatible with various precursors. The radionuclidic purity was 100%, and the radiochemical purity remained consistently above 95%. Moreover, the radiochemical yield ranged from 69% to 83%, which ensured the efficient utilization of high-activity ^68^Ga.

**Table 2:** Examples of ^68^Ga-labeled precursor synthesis conditions and quality control results (*n* = 3)

^68^Ga-labeled precursor	[^68^Ga]Ga-DOTA-TATE	[^68^Ga]Ga-Pentixafor	[^68^Ga]Ga-FAP-2286
Peptide amount	50 µg	50 µg	50 µg
Buffer	Sodium acetate	Sodium acetate	Sodium acetate
Labeling conditions	95 °C, 5 min, pH = 4.4	95 °C, 10 min, pH = 4.4	95 °C, 5 min, pH = 4.4
Appearance	Colorless, no particles	Colorless, no particles	Colorless, no particles
Radionuclidic purity	100%	100%	100%
Radiochemical purity	99.8 ± 0.2%	98.6 ± 0.4%	98.9 ± 0.7%
Radiochemical yield (Decay corrected)	81 ± 3%	83 ± 5%	69 ± 4%
Stability (2 h, RCP)	99.5 ± 0.2%	97.2 ± 0.3%	95.7 ± 0.5%

## Discussion

The critical parameters of the ^68^Ge/^68^Ga generator include ^68^Ga elution yield and ^68^Ge breakthrough, which are generally challenging to balance. The details of commercially available ^68^Ge/^68^Ga generators were summarized in Table 3 (Cressier et al. 2015; Amor-Coarasa et al. 2016; Jernström et al. 2017; Fialová et al. 2024; Feng et al. 2025). As can be seen in Table 3, SnO_2_-based and pyrogallol-derivatized SiO_2_-based generators have high elution yield (> 80%) but show a relatively higher ^68^Ge breakthrough, approximately 0.001% for SnO_2_ and 0.005% for pyrogallol-derivatized SiO_2_. However, SnO_2_-based and pyrogallol-derivatized SiO_2_-based generators face challenges in obtaining approval from the EMA and FDA due to their relatively high ^68^Ge breakthrough levels. In contrast, TiO_2_-based generators, which have lower elution yields, exhibit a lower ^68^Ge breakthrough (< 0.001%), and have been successfully approved by the EMA and FDA. Besides, increasing the ^68^Ge loading capacity is difficult, as higher loading activity may reduce elution yield and increase ^68^Ge breakthrough (Waterhouse et al. 2020). Here, a novel modified TiO_2_-based ^68^Ge/^68^Ga generator with an activity of 3.26 GBq was presented. This generator demonstrates high elution yield over the two-year evaluation period, with average ^68^Ga elution yield around 80%, and a low ^68^Ge breakthrough rate at the 0.00003% level, which is more than 30-fold lower than the limit set by the European Pharmacopoeia. Moreover, the low metal impurity levels eliminate the need for additional purification, and the NRT generator is capable of employing a small-volume elution method to attain a higher activity concentration.

**Table 3:** Comparison between generator characteristics

Generator	Sorbent	C_HCl_	V_el_	^68^Ga elution yield		^68^Ge breakthrough
				Initial	Long term	
Cyclotron Co., Ltd (Obninsk)	TiO_2_	0.1 M	5 mL	> 80%	45% (3 years)	≤ 0.005%
Eckert & Ziegler (IGG100)	TiO_2_	0.1 M	5 mL	> 70%	60% (1 year)	< 0.001%
Eckert & Ziegler (GalliaPharm^®^)	TiO_2_	0.1 M	5 mL	> 70%	60% (1.5 year)	< 0.001%
IRE EliT (Galli Ad™)	TiO_2_	0.1 M	1.1 mL	70%	55% (1 year)	< 0.001%
NRT generator	TiO_2_	0.1 M	5 mL	> 80%	70% (2 year)	< 0.001%
iThemba LABS	SnO_2_	0.6 M	6 mL	> 80%	75% (300 days)	~ 0.001% (≤ 0.002%)
Pars Isotope	SnO_2_/TiO_2_	0.1 M	3 mL	70%	50% (270 days)	< 0.001%
ITG GmbH	modif. SiO_2_	0.05 M	4 mL	> 80%	70% (1 year)	≤ 0.005%
ITG GmbH	Ta_2_O_5_	0.05 M	4 mL	> 70%	60% (1 year)	< 0.001%

It is worth noting the influence of room temperature on the ^68^Ga elution yield of the NRT generator. As can be seen in Table 4; Fig. 3, at the two low temperature points (the 245th and 576th days) within the two-year period, the ^68^Ga elution yields were the lowest, at 72.8% and 73.3% respectively. This is likely attributable to the lower temperature, which suppresses the free diffusion of ^68^Ga^3+^ free ions and thereby hinders their elution from the sorbent into the HCl solution. When the room temperature is increased, the high elution yield is restored, indicating that low temperatures do not damage the generator. Thus, the NRT ^68^Ge/^68^Ga generator is more suitable for elution at an ambient temperature above 20 °C to obtain higher ^68^Ga activity. Furthermore, multiple daily elutions of the generator can maximize the utilization of the ^68^Ge/^68^Ga generator. The theoretical ingrowth curve of ^68^Ga from a ^68^Ge/^68^Ga generator has been described previously (Roesch 2012), as shown in Fig. S2, following the growths of ^68^Ga activity on the generator column, 50% of the theoretical maximum is reached within one half-life. After more than three half-lives (about 3.8 h), 90% of the maximum value is achieved. Consequently, the generator can be eluted multiple times per day, and an example of the NRT generator is presented in Table S1.

**Table 4:** Selected data comparison for ^68^Ga elution yield and ^68^Ge breakthrough

Date (day)	1st	181st	245th	346th	576th	731st
Room temperature (°C)	24.6	20.1	15.4	22.9	11.4	23.4
^68^Ge activity (mCi)	87.7	55.4	47.0	36.3	20.2	13.6
^68^Ga eluate activity (mCi)	75.2	44.3	34.2	29.7	14.8	11.0
Elution yield (%)	85.7	80.0	72.8	81.8	73.3	80.9
^68^Ge breakthrough (%)	0.000027	0.000046	0.000014	0.000021	0.000009	0.000031

The NRT generator achieves a high ⁶⁸Ge loading capacity of 3.26 GBq, a high ^68^Ga elution yield, and minimal ^68^Ge breakthrough, which may be attributed to the structural and morphological advantages of the home-made TiO_2_ sorbent (as shown in Fig. S3), including a denser structure, a more stable and smoother surface, radiation resistance and an absence of readily dislodged fine particles (Dash and Chakravarty 2017).

In summary, a higher ^68^Ga elution efficiency enables a more effective utilization of the expensive ^68^Ge raw material, thereby maximizing the production of ^68^Ga. Additionally, a higher ^68^Ge loading capacity and a longer shelf life can maximize the utilization of radionuclides and minimize the generation of waste. This reduces the replacement frequency of the costly ^68^Ge/^68^Ga generator and decreases economic expenditure, which is in high accordance with the principles of green radiochemistry and sustainable radiopharmaceutical production. In the future, further research and development are required to improve its applicability, including achieving GMP-compliant production and obtaining EMA or FDA approval.

## Conclusion

It is feasible to develop a ^68^Ge/^68^Ga generator that combines high ^68^Ga elution yield with minimal ^68^Ge breakthrough. Furthermore, the ^68^Ge loading activity has been increased to 3.26 GBq (88 mCi). This generator demonstrates excellent performance characteristics over a two-year period. It significantly advances the principles of green radiochemistry and promotes sustainable and environmentally friendly radionuclide production.

## Supplementary Information

Below is the link to the electronic supplementary material.

Supplementary Material 1

## Data Availability

The datasets used and/or analysed during the current study are available from the corresponding author on reasonable request.

## Abbreviations

EMA: European Medicines Agency
FDA: American Food and Drug Administration
ICP-OES: Inductively coupled plasma optical emission spectrometry
Ph. Eur.: European Pharmacopoeia
TLC: Thin-layer chromatography
DOTA: 1,4,7,10-Tetraazacyclododecane-1,4,7,10-tetraacetic acid
HPLC: High performance liquid chromatography
GMP: Good manufacturing practice
