# Supplementary material for: Long-term performance evaluation of a novel 3.26 GBq 68Ge/68Ga generator
Source: EJNMMI Radiopharm Chem. 2026 Mar 1;11:24. doi: 10.1186/s41181-026-00429-z (PMC13057101; doi:10.1186/s41181-026-00429-z)
Supplement: Supplementary file 1 — Supplementary Material 1 [file 41181_2026_429_MOESM1_ESM.docx]

**Supplementary Information**

**Long-term performance** **evaluation of a novel 3.26 GBq ^68^Ge/^68^Ga generator**

Xiaoyang Hu^1,2^, Renxin Hu^2^, Yao Yang^2^, Yuping Li^2^, Yan Zhao^3^, Ning Liu^4^, Qiang Ge^2^, Shuang Zhang^2,4*^, Songdong Ding^1*^

1. College of Chemistry, Sichuan University, Chengdu, China

2. Chengdu New Radiomedicine Technology Co., Ltd., Chengdu, China

3. Department of Nuclear Medicine, Affiliated Hospital of Southwest Medical University, Luzhou, China

4. Institute of Nuclear Science and Technology, Sichuan University, Chengdu, China

**Corresponding author:**

Professor Songdong Ding

College of Chemistry

Sichuan University

Chengdu 610064, China

Office: +86 28 85412329 (S. D. Ding)

Email: dsd68@163.com (S. D. Ding), shuang.zhang@nrtmedtech.com (S. Zhang)

**1. Radiochemical identity of [^68^Ga]GaCl_3_**

Thin-layer chromatography (TLC) was used to determine the radiochemical identity and radiochemical purity of [^68^Ga]GaCl_3_, using a radio-TLC scanner (Mini-scan, Eckert & Ziegler, Germany). Plate: TLC silica gel plate (ITLC-SG, 1.5×20 cm, Agilent Technologies, USA); mobile phase: 77 g/L solution of ammonium acetate and methanol (50:50, *V*/*V*). Development: immediately, over a path of at least 10 cm.

Test solution: take an appropriate volume of [^68^Ga]GaCl_3_ and dilute it with 0.1 M HCl to a concentration of approximately 37 MBq/mL. Subsequently, pipette 0.3 mL of this prepared solution and add 0.3 mL of 0.5 mol/L HCl solution.

Reference solution (a): to 0.2 mL of the test solution, add 0.3 mL of a 4 g/L solution of sodium hydroxide. Use within 30 min of preparation.

Reference solution (b): to 0.2 mL of the test solution, add 0.2 mL of a 10 g/L solution of pentetic acid in 4 g/L solution of sodium hydroxide. Use within 30 min of preparation.


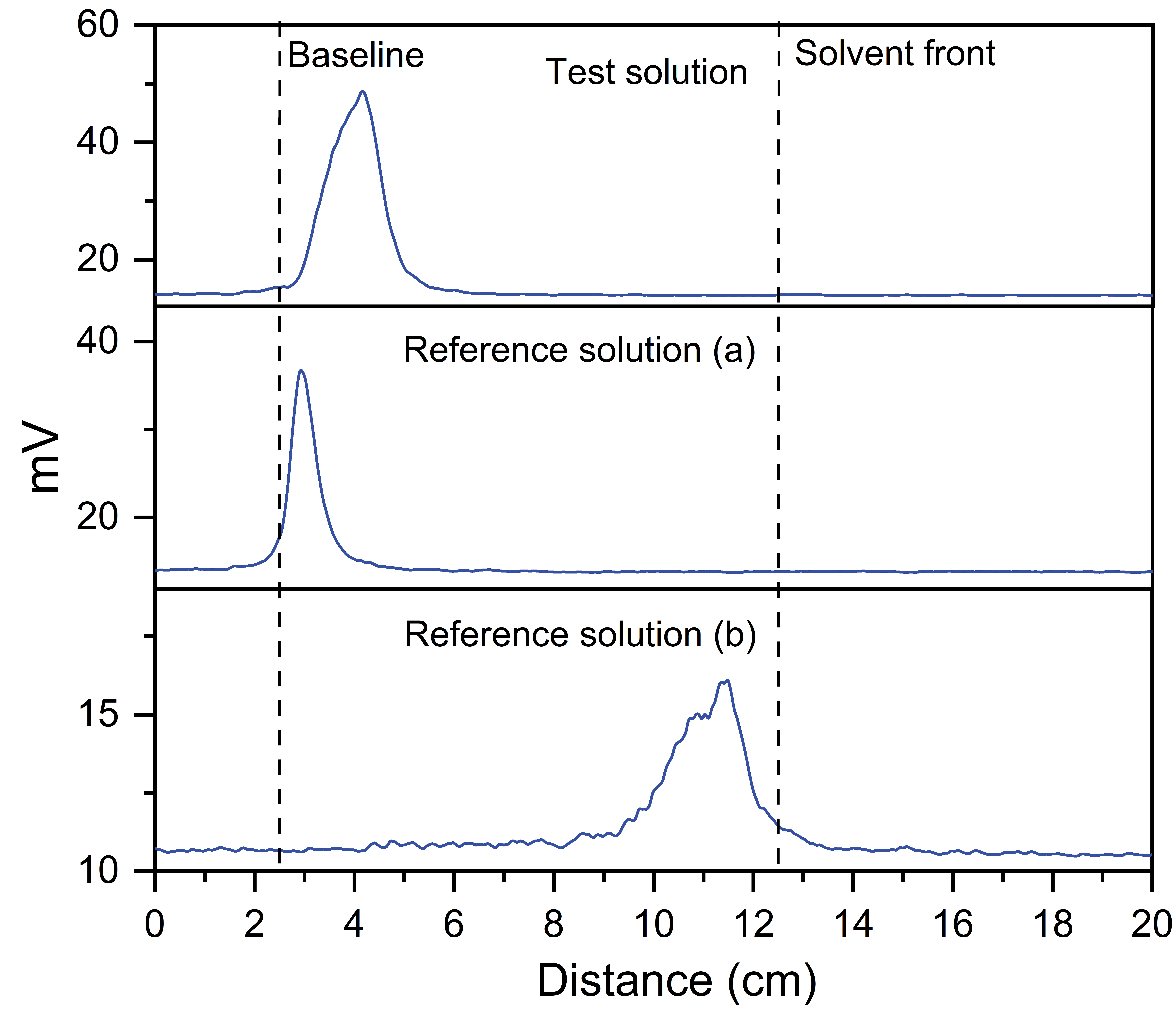


**Fig. S1** Radio-TLC chromatograms of [^68^Ga]GaCl_3_

**2.** **The theoretical ingrowth curve of ^68^Ga from a ^68^Ge/^68^Ga generator**


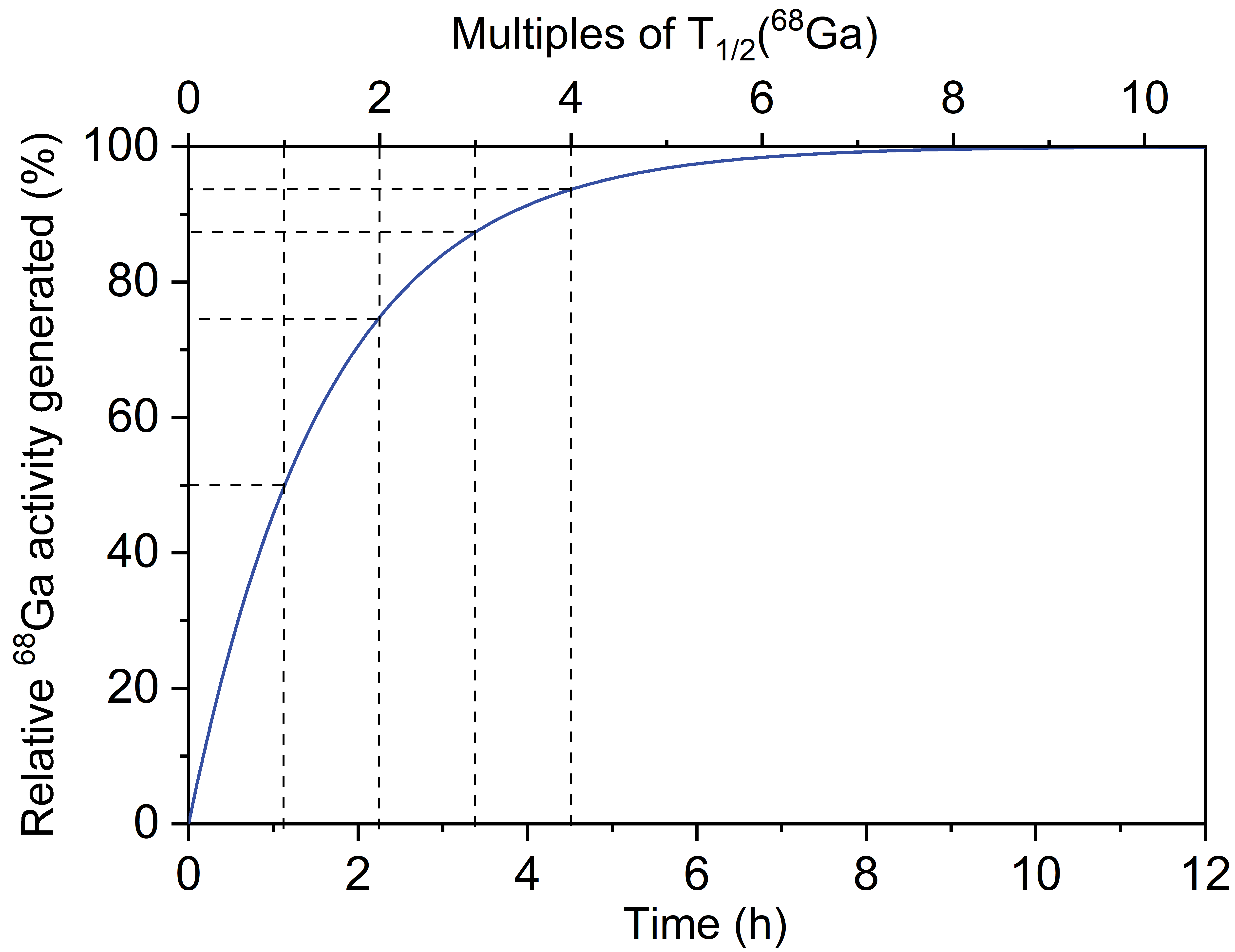


**Fig. S2** Generation kinetics of ^68^Ga on the generator column following an initial elution. Upper x-axis: number of half-lives of ^68^Ga.

The ^68^Ga build-up activity can be calculated by the Eq. S1:

|  | ${}^{\text{68}}\text{Ga}\text{ build-up activity}=A_{\mathrm{Ge}}\times(1-e^{-\lambda_{\mathrm{Ga}}\times t})$ | (S1) |
| --- | --- | --- |

where *A*_Ge_ is the activity of the ^68^Ge/^68^Ga generator. *λ*_Ga_ represents the decay constant (*λ* = ln2/*T*_1/2_ = 0.0102 min^−1^) of ^68^Ga, and *t* is the time interval between two elutions.

The Table S1 provides an example. According to the actual situation and the required activity level, the NRT ^68^Ge-^68^Ga generator can be eluted multiple times a day.

**Table S1** Multiple eluations of the NRT ^68^Ge/^68^Ga generator on the 321st day

| No. | 1st | 2nd | 3rd |
| --- | --- | --- | --- |
| Interval time | 20h | 3h50min | 2h |
| ^68^Ge activity | 38.7 mCi | 38.7 mCi | 38.7 mCi |
| ^68^Ga build-up activity | 38.7 mCi | 34.8 mCi | 27.5 mCi |
| ^68^Ga eluant activity | 30.5 mCi | 27.9 mCi | 21.8 mCi |
| Elution yield | 78.8% | 80.2% | 79.3% |

**3. SEM image of the modified TiO_2_ sorbent**


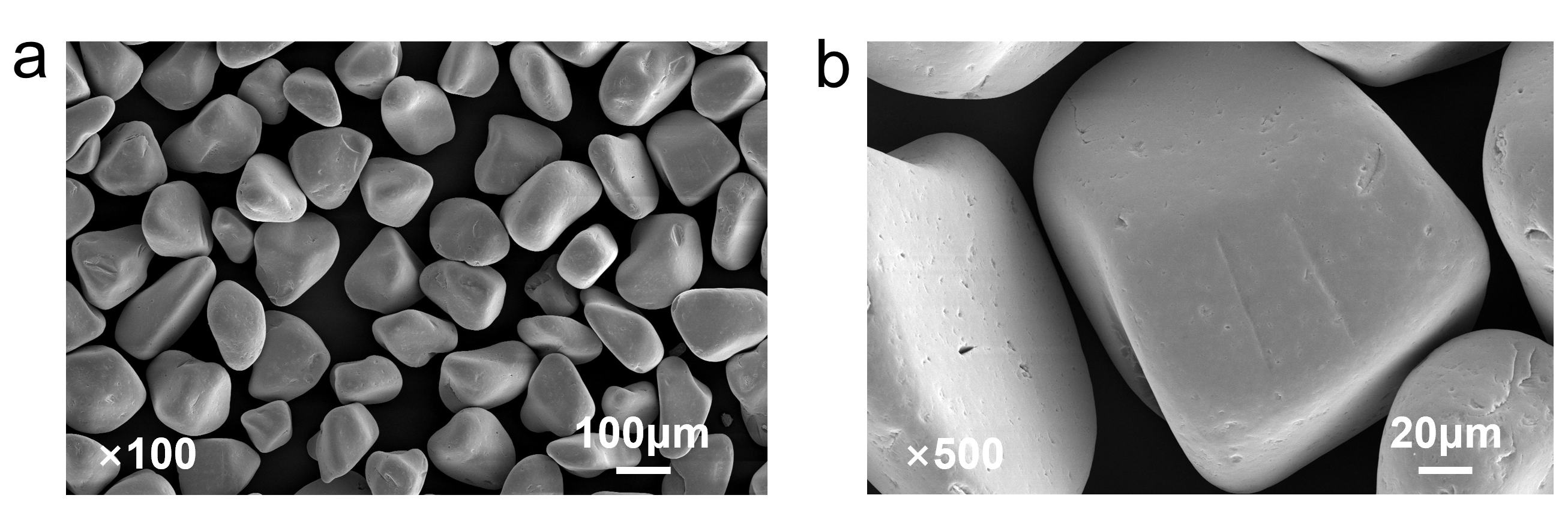


**Fig. S3** SEM image of the modified TiO_2_ sorbent

**4. Radiochemical purity of [^68^Ga]Ga-DOTA-TATE**

Radiochemical purity of [^68^Ga]Ga-DOTA-TATE was determined using Shimadzu LC-2030C HPLC System (Shimadzu, Japan) with a Flowcount Pro radiation detector (Eckert&Ziegler, Germany). Column: Agilent SB-C18 4.6 mm × 250 mm, 5 μm; mobile phase: water/0.1% trifluoroacetic acid (TFA) (A) and acetonitrile/0.1% TFA (B): 0–15 min, 10%–90% B; 15–20 min, 90% B; flow rate: 1 mL/min.


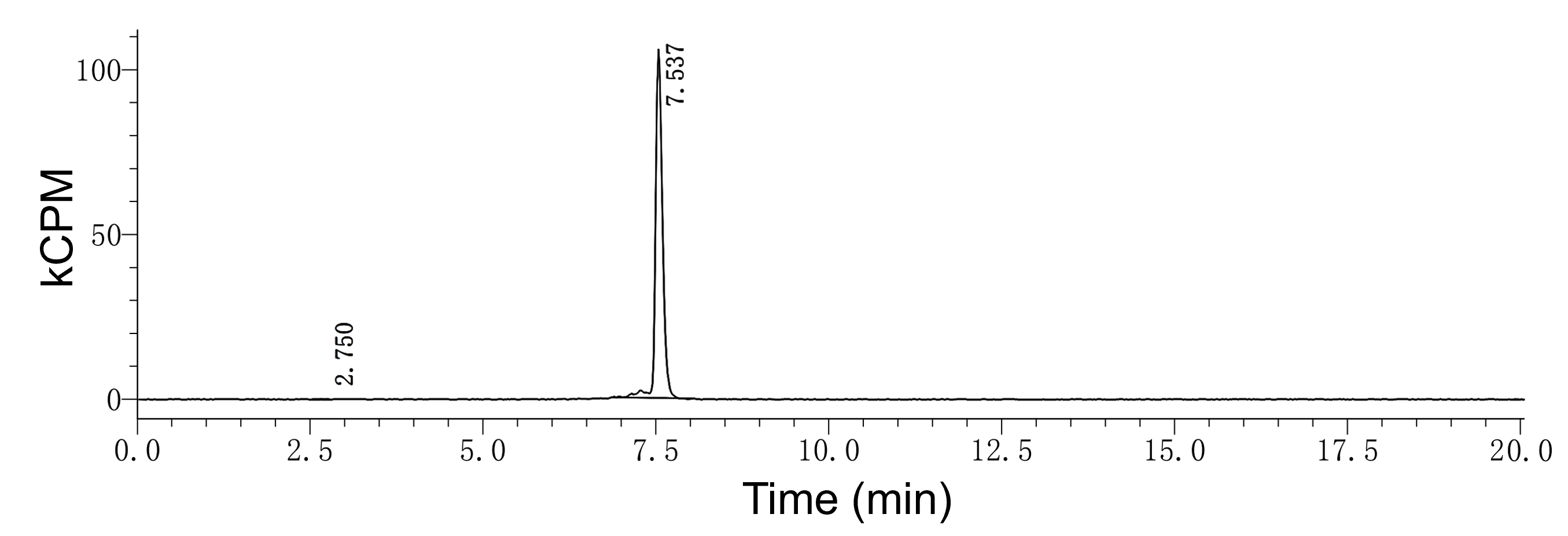


**Fig. S4** Radio-HPLC chromatogram of [^68^Ga]Ga-DOTA-TATE

**5. Radiochemical purity of [^68^Ga]Ga-Pentixafor**

Radiochemical purity of [^68^Ga]Ga-Pentixafor was determined using Agilent 1260 Infinity II System (Agilent Technologies, USA) with a Flow Star LB 514 radiation detector (Berthold Technologies, Germany); Column: zhongpu science RX-C18, 4.6 × 50 mm, 3 μm; mobile phase: water/0.1% TFA (A) and acetonitrile/0.1% TFA (B): 0–10 min, 10%–90% B; flow rate: 0.8 mL/min.


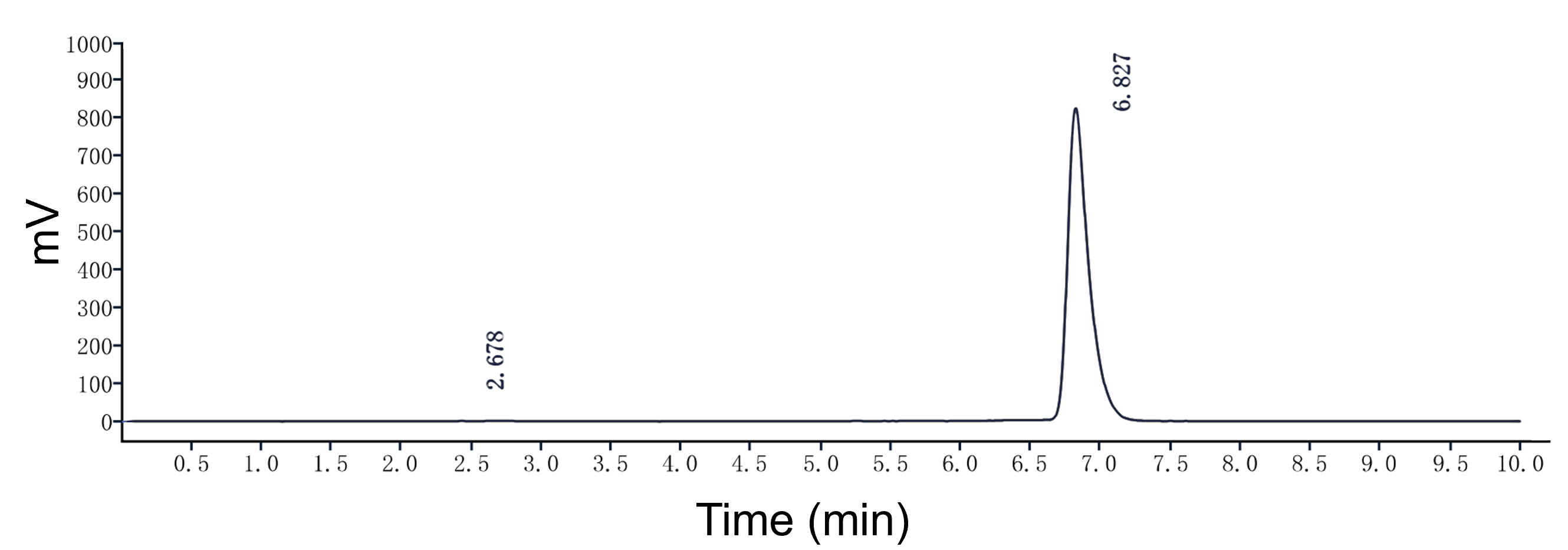


**Fig. S5** Radio-HPLC chromatogram of [^68^Ga]Ga-Pentixafor

**6. Radiochemical purity of [^68^Ga]Ga-FAP-2286**

Radiochemical purity of [^68^Ga]Ga-FAP-2286 was determined using Shimadzu LC-2030C HPLC System (Shimadzu, Japan) with a Flowcount Pro radiation detector (Eckert&Ziegler, Germany). Column: Agilent SB-C18 4.6 mm × 250 mm, 5 μm; mobile phase: water/0.1% TFA (A) and acetonitrile/0.1% TFA (B): 0–15 min, 10%–90% B; 15–20 min, 90% B; flow rate: 1 mL/min.


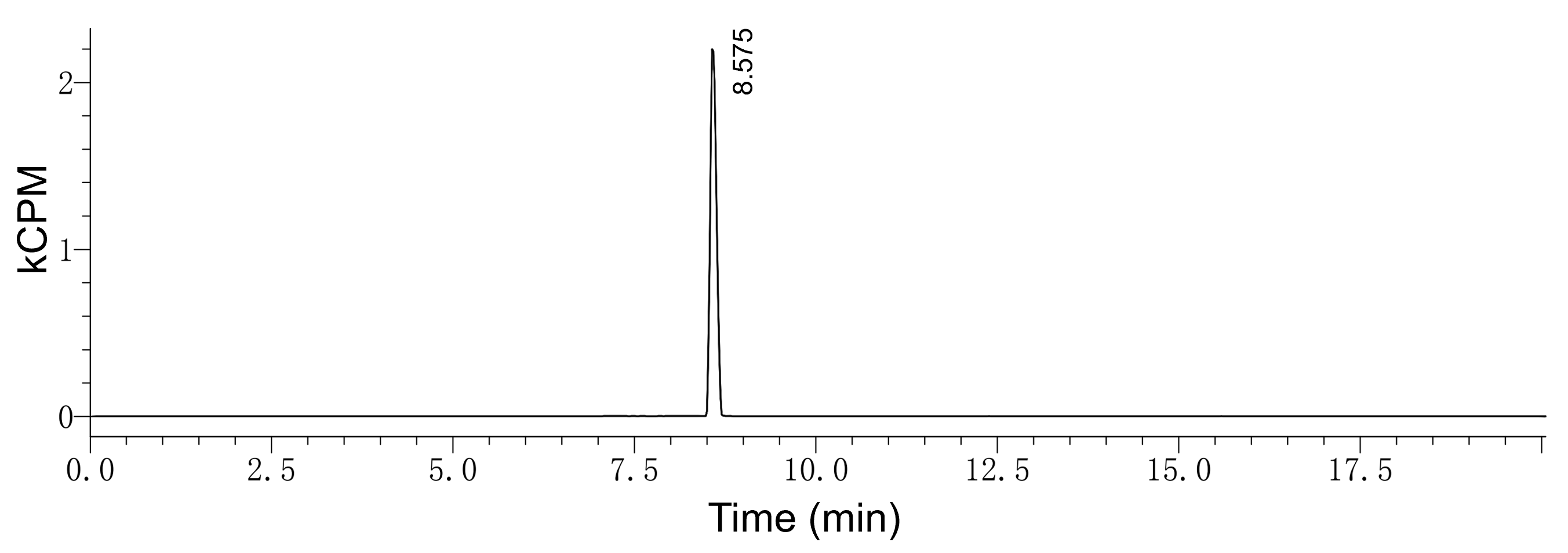


**Fig. S6** Radio-HPLC chromatogram of [^68^Ga]Ga-FAP-2286
